# Potential Association between Vaginal Microbiota and Cervical Carcinogenesis in Korean Women: A Cohort Study

**DOI:** 10.3390/microorganisms9020294

**Published:** 2021-01-31

**Authors:** Gi-Ung Kang, Da-Ryung Jung, Yoon Hee Lee, Se Young Jeon, Hyung Soo Han, Gun Oh Chong, Jae-Ho Shin

**Affiliations:** 1 Department of Applied Biosciences, Kyungpook National University, Daegu 41566, Korea; gukang@knu.ac.kr; 2 Department of Biomedical Convergence Science & Technology, Kyungpook National University, Daegu 41566, Korea; amugae1210@knu.ac.kr; 3 Department of Obstetrics and Gynecology, School of Medicine, Kyungpook National University, Daegu 41404, Korea; mylyh3@naver.com (Y.H.L.); tpqkf0927@naver.com (S.Y.J.); 4 Department of Obstetrics and Gynecology, Kyungpook National University Chilgok Hospital, Daegu 41404, Korea; 5 Department of Physiology, School of Medicine, Kyungpook National University, Daegu 41405, Korea; hshan@knu.ac.kr

**Keywords:** vaginal microbiome, CIN severity, CIN prediction, vaginosis

## Abstract

Convincing studies demonstrated that vaginal flora is one of the most impactful key components for the well-being of the genital tract in women. Nevertheless, the potential capability of vaginal-derived bacterial communities as biomarkers to monitor cervical carcinogenesis (CC) has yet to be studied actively compared to those of bacterial vaginosis (BV). We hypothesized that vaginal microbiota might be associated with the progression of CC. In this study, we enrolled 23 participants, including healthy controls (HC group; n = 7), patients with cervical intraepithelial neoplasia (CIN) 2 and 3 (CIN group, n = 8), and patients with invasive cervical cancer (CAN group; n = 8). Amplicon sequencing was performed using the Ion Torrent PGM to characterize the vaginal microbiota. Patients with CIN and CAN presented vaginal microbiota dysbiosis compared with HC. The alpha diversity analysis revealed that CC has a trend to be increased in terms of diversity indexes. Moreover, CC was associated with the abundance of specific microbes, of which *Lactobacillus* and *Gardnerella* were the most significantly different between HC and CIN, whereas *Streptococcus* was differentially abundant in CAN compared with CIN. We then evaluated their diagnostic abilities. Testing in terms of diagnostic ability using the three genera revealed considerably high performance with an area under the receiver-operating characteristic curve of 0.982, 0.953, and 0.922. The current study suggests that the presence of *Gardnerella* and *Streptococcus* may be involved in the advancment of CC.

## 1. Introduction

Compelling studies revealed that persistent infection with certain types of the oncogenic virus, known as human papillomavirus (HPV), is a necessary cause of the progress of cervical intraepithelial neoplasia (CIN) and invasive cervical cancer (ICC) [1,2,3]. Although there is no doubt that HPV infection is the primary cause, data regarding whether the virus drives full tumorigenesis is still insufficient. Kyrgiou et al. [4] reported that cervicovaginal bacterial composition may influence the presence of subsequent cervical preinvasive disease.

The indigenous vaginal microbial ecosystem—known as the microbiota—is composed of microorganisms that can strongly affect gynecological wellness and women’s health, being involved in the metabolic process [5] and immunological response [6,7]. Among the microbiota, *Lactobacillus* spp., which produce lactic acid [8] and bacteriocins [9], are the key microbes in healthy women and involved in creating the stability of the vaginal microbial composition, maintaining a low-pH environment [10,11]. Thus, the *Lactobacillus*-dominated environment is considered a symbol of vaginal health. With the development of next-generation sequencing that revolutionized traditional microbiology, Ravel et al. [12] categorized vaginal microbial community into five community state types (CSTs) at the species-level resolution using amplicon sequencing. In the classification, CSTs I, II, III, and V were defined by the high relative abundance of *Lactobacillus crispatus*, *Lactobacillus gasseri*, *Lactobacillus iners*, and *Lactobacillus jensenii*, whereas CST IV was defined by the low abundance of *Lactobacillus* with bacteria such as *Gardnerella*, *Streptococcus*, *Prevotella*, and *Sneathia*.

Recent systematic reviews demonstrated that the disturbances of vaginal microbiota are possibly associated with bacterial vaginosis (BV), human papillomavirus (HPV) infection, and tubal factor infertility [13,14,15]. In addition, changes in the vaginal microenvironment along the genital tract in women are possibly correlated to vulvovaginal candidiasis, sexually transmitted diseases, preterm birth, and so on [16,17,18,19]. Moreover, Mitra et al. [20] reported that the CIN disease severity might be associated with the increase of vaginal microbiota diversity. In the context of the studies that showed the clinical and research implications of vaginal microbiota, we hypothesized that the microbiota might be associated with cervical carcinogenesis (CC). Therefore, the purpose of this study was to explore the potential association of vaginal microbial composition with CC and present a diagnostic usefulness for CC progression, prediction, and classification, which distinguish diseased samples from healthy controls.

## 2. Materials and Methods

### 2.1. Study Cohort

The ethical approval of the present study was obtained from the institutional review board of Kyungpook National University Chilgok Hospital (KNUMC 2015-10-033, 16-11-2015). All the procedures were performed according to the Declaration of Helsinki. For this study, we recruited 23 women and divided them into 4 groups as follows: healthy controls (HC group, n = 7), patients with CIN 2 and 3 (CIN group, n = 8), and ICC (CAN group, n = 8). Vaginal smears were taken using pap brush-lines (Bion, Korea) from healthy women and patients with CIN and ICC. All collected vaginal samples were sent to the local laboratory, and immediately moved to DNase, RNase, and pyrogenic-free tubes.

### 2.2. HPV Assay and HPV Genotyping

Cervicovaginal swab specimens were served to perform the genotyping of HPV infection using the Anyplex II HPV 28 assay kit (Seegene, Korea). The HPV assay based on real-time PCR was conducted according to the construction of manufacturer. A total of 28 HPV subtypes were targeted to detect high-risk HPV (16, 18, 26, 31, 33, 35, 39, 45, 51, 52, 56, 58, 59, 66, 68, 69, 73, and 82) and low-risk HPV (6, 11, 40, 42, 44, 53, 54, and 70).

### 2.3. DNA Extraction and High-Throughput Sequencing

Total bacterial DNA extraction was performed using the QIAamp PowerSoil Pro DNA Kit (QIAGEN, Hilden, Germany) according to the protocol of the manufacturer. For the preparation of the sequencing library, PCR amplification was performed using primer pairs targeting the V3 region of the 16S rRNA gene, 338F (5′-barcode-ACTCCTACGGGAGGCAGC-3′), and 534R (5′-barcode-ATTACCGCGGCTGCTGG-3′), as described previously [7]. Then, PCR products were pooled in equal concentrations, and the concentration of bacterial DNA was measured using a Qubit 3.0 Fluorometer (Invitrogen, Carlsbad, CA, USA). The final products were sequenced in the Ion Torrent PGM for 1250 flows with the Ion PGM Hi Q Sequencing Kit (Thermo Fisher, Waltham, MA, USA), following the manufacturer’s instruction.

### 2.4. Bioinformatic Analysis

For the analysis of 16S rRNA gene sequencing, the raw sequencing reads were obtained from the Ion Torrent Software Suite as the FASTQ files and the Quantitative Insights into Microbial Ecology 2 (QIIME2) v. 2020.8 software [21] was used for further processing after removal of the adapter sequences. To produce amplicon sequence variants (ASV), the DADA2 software [22] was used for quality filtration (mean frequency of 17,349), trimming, and denoizing (Q score > 30). We then removed the ASVs that did not belong to bacterial sequences (non-bacterial, mitochondrial, and chloroplast sequences), and the mean sample depth filtered was <0.1%. SILVA v138 with 99% cutoff value as a reference database was served for assigning taxonomic identities to each ASV at various taxonomic levels. To normalize the different sequencing depths from each sample, sequences among the samples were rarefied to a sequencing depth of 6919 reads. Subsampling of the feature tables in every sample was performed at equal depths for the downstream analysis.

### 2.5. Statistical Analysis

Statistical analyses and visualization of amplicon sequencing data were performed using RStudio 4.0.3 (https://www.rstudio.com/). The alpha diversity indexes (Shannon, Richness, and Simpson’s index) were computed using the vegan package [23], and the Kruskal–Wallis test was applied to evaluate the statistical significance. Beta diversity to measure the differences between groups was performed as the principal coordinate analysis (PCoA), based on the Bray–Curtis dissimilarity. An Adonis test was applied to investigate pairwise comparisons of each group. The heat map of the top 25 genera of each health status was created using the gplots package [24]. To investigate the biomarker that characterizes the microbial differences between different health statuses, the LDA effect size (LEfSe) was performed on the basis of the Galaxy Web application and workflow framework (https://huttenhower.sph.harvard.edu/galaxy/). In this analysis, the factorial Kruskal–Wallis and pairwise Wilcoxon tests were applied with an alpha value of 0.05 to detect significant features between the classes. Then, only LDA values >7.0 were presented. The diagnostic performance of the prediction model based on the biomarkers was then assessed and calculated using the area under the receiver-operating characteristic (ROC) curve (AUC) in the pROC package [25]. The corrplot package of the R software was used to compute and display the values [26].

## 3. Results

### 3.1. Participants’ Characteristics

Twenty-three women were enrolled in this study. The HC group accounted for seven women; the CIN group, for eight women; and the CAN group, for eight women. The healthy group was defined as women who had normal cervical pathology. Exclusion criteria included women with unknown HPV infection status, no symptoms of vaginitis such as vaginal itching sense or foul odor discharge, and without sexually transmitted disease. Additionally, women who had other malignancies confirmed in cervical pathology and treated using antibiotics or vaginal tablets were further excluded. The basic characteristics of each group are shown in Table 1. The CIN and CAN groups had high prevalence rates of HPV infection, wherein most of the subjects in the CAN group had high-risk HPV and all patients in the CIN group had low risks related to the HPV type. The mean age of the HC group showed the highest among all groups.

### 3.2. Vaginal Microbiota in Disease Progression

**Figure 1:**
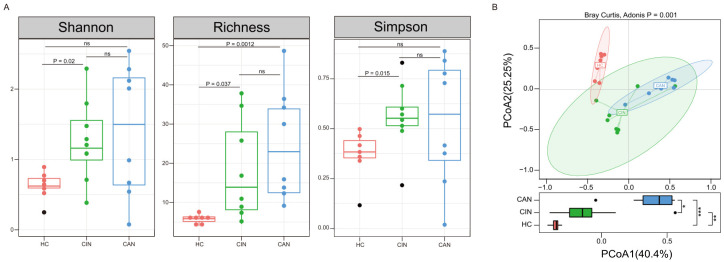
The differences in vaginal microbiota diversity by health status. (**A**) The boxplots display the differences in the alpha diversity indexes (Shannon index: left panel, Richness: middle panel, and Simpson’s index: right panel) between the groups. (**B**) Principal coordinate analysis (PCoA) plot depending on the Bray–Curtis dissimilarity of beta diversity, which colored each sample according to health status. The HC group shows lower inter-individual variations than the CIN and CAN groups at the PCoA axis1; * *p* < 0.05, ** *p* < 0.01, *** *p* < 0.001.

To determine if disease progression affect the vaginal microbial diversity, we compared the dynamics of the microbiota by each health status. Both alpha diversity indexes and richness at the genus level were significantly lower in the HC group than those in CIN and CAN groups (Figure 1A). Although statistical significance was not reached, increasing trends with disease severity were observed with the lowest indexes in the HC group and highest indexes in the CAN group in the alpha diversity analysis. Beta diversity was computed using a two-dimensional PCoA depending on the Bray–Curtis dissimilarity to estimate differences of microbial structure of each group. The vaginal microbiota compositions of the participants were significantly different (Adonis, *p* = 0.001; Figure 1B). The complicated alterations in the vaginal microbial composition of each health status seemed to occur during severity progression from a healthy status to cervical cancer stage. To evaluate the statistical significance of the trend, we further calculated the dispersion among health statuses. The first axis of the PCoA (40.4% of the total variation) capturing one of the major causes of microbial variation was potentially associated with disease progression, suggesting that microbial changes are distinguishable for disease severity.

### 3.3. Comparative Analysis of the Vaginal Microbiota between the Groups

**Figure 2:**
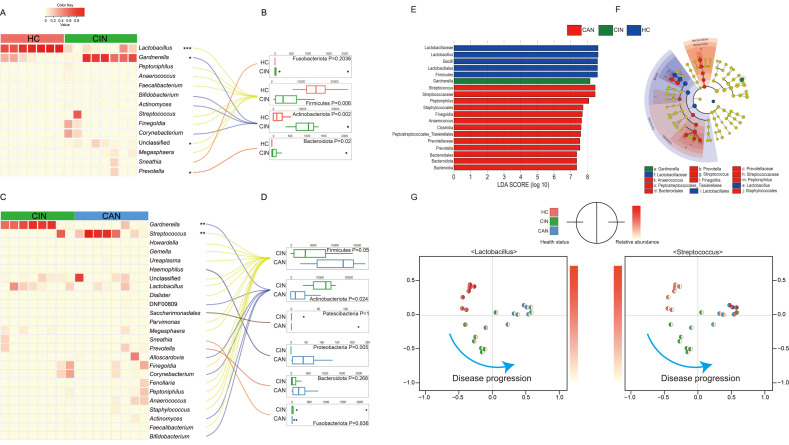
Comparative analysis of vaginal microbiota profiles. Differences in microbial composition between each group at the genus (**A**,**C**) and corresponding phylum levels (**B**,**D**) are presented as a heat map. The LDA effect size (LEfSe) analysis to identify the potential biomarkers revealed changes of the vaginal microbiota according to health status (**E**,**F**). (**G**) The Bray–Curtis dissimilarity based PCoA plot is colored by the relative abundance of the *Lactobacillus* and *Streptococcus* genera; the left hemisphere indicates the health status and the right presents the relative abundance of each bacterial genus. Statistical significance was computed using the Wilcoxon rank–sum test; * *p* < 0.05, ** *p* < 0.01, *** *p* < 0.001.

Previous studies revealed that the vaginal microbiota can be affected and altered by health status in the vaginal tract [27,28,29,30]. Here, we evaluated differences in taxonomic abundances at the phylum and genus levels (Figure 2). When sequences were assigned to genus level, we observed microbial shifts in four genera, with three genera abundant in the CIN group and only one genus abundant in the HC group (Figure 2A). Among the genera, *Lactobacillus* was significantly more abundant in the HC group (*p* < 0.001), whereas *Gardnerella*, Unclassified, and *Prevotella* were abundant in the CIN group (*p* = 0.023, *p* = 0.024, and *p* = 0.019, respectively). The abundance of Firmicutes (*p* = 0.006) was markedly higher, while significant reductions in the Bacteroidota (*p* = 0.02) and Actinobacteriota (*p* = 0.002) phyla were found in the HC group than in the CIN group at the phylum level (Figure 2B). In the comparison of the CIN and CAN groups, *Gardnerella* (*p* = 0.002) and *Streptococcus* (*p* = 0.004) were the only microorganisms that differed significantly between each group, with the former dominant in the CIN group and the latter dominant in the CAN group (Figure 2C). In addition, the CAN group showed increased Firmicutes and Proteobacteria compared with the CIN group at the phylum level (Figure 2D). To identify the potential bacterial candidates as biomarkers associated with disease severity, LDA effect size (LEfSe) analysis was performed at various taxonomic levels. Microbial taxa with the LDA score >7.0 and *p* < 0.05 are considered potential biomarkers in each group, and this showed a consistent trend as in the above-described abundance difference analysis (Figure 2). *Lactobacillus* was chosen as the most representative genus in the HC group; *Gardnerella*, in the CIN group; and *Streptococcus*, in the CAN group (Figure 2E,F). To determine if the representative microbes can be considered potential biomarkers, we colored each sample with their relative abundance, indicating that the difference in health status could be elucidated based on the relative abundance of *Lactobacillus* and *Streptococcus* genera in each group (Figure 2G).

### 3.4. Predictive Ability of the Proposed Biomarkers

**Figure 3:**
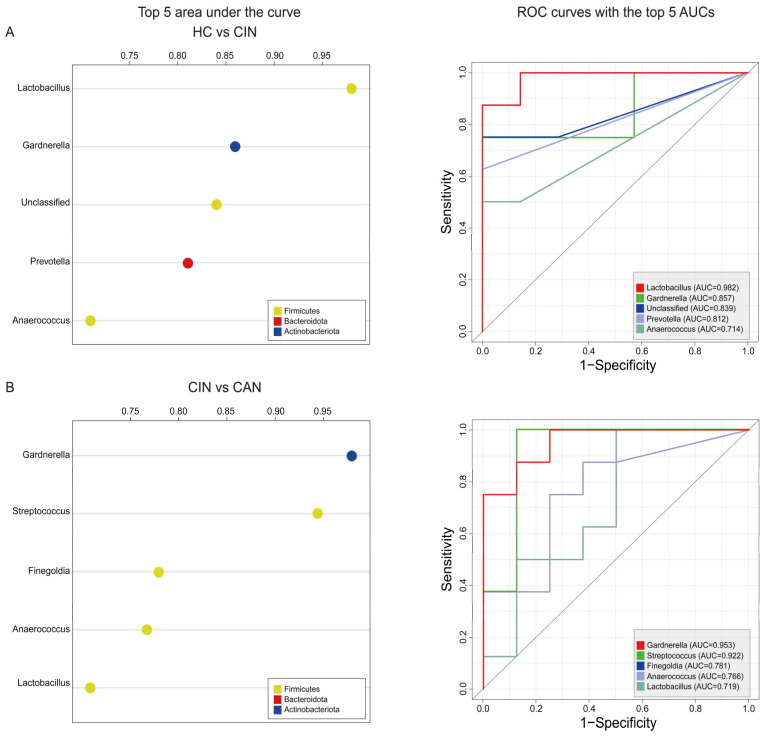
Evaluation of the top five bacterial genera as a noninvasive diagnostic tool to predict disease progression. (**A**) ROC analysis to compare HC with CIN. (**B**) ROC analysis to compare CIN with CAN. The left panel displays the AUC of the top five impactful genera, and the right panel presents the individual AUC of the ROC curves.

We then conducted the area under the ROC curve analysis using samples from the HC and CIN groups to examine the potential effects of biomarkers in terms of diagnosis. We observed that five bacterial genera, namely, *Lactobacillus*, *Gardnerella*, Unclassified, *Prevotella*, and *Anaerococcus*, had the highest discriminatory value (Figure 3A). Among the five genera, *Lactobacillus* showed the strongest diagnostic power (AUC = 0.982), followed by *Gardnerella* (AUC = 0.857, Figure 3B). When the CIN group was compared with the CAN group, we found that *Gardnerella*, *Streptococcus*, *Finegoldia*, *Anaerococcus*, and *Lactobacillus* are the most impactful factors to discriminate CAN from CIN as shown in Figure 3B. Notably, the discriminators of disease severity were the same microbes as identified in the LEfSe analysis. These data suggest that disease severity led to depletion and difference in microbes. Taken together, the AUC scores based on the ROC analysis revealed that two microbes in each model had AUC scores >0.80, indicating that the progression of disease alters the vaginal microbial composition, and these proposed microbes can accurately distinguish the severity of the disease in both cases (HC vs. CIN and CIN vs. CAN).

### 3.5. The Impact of HPV Infection across the Vaginal Microbiota

**Figure 4:**
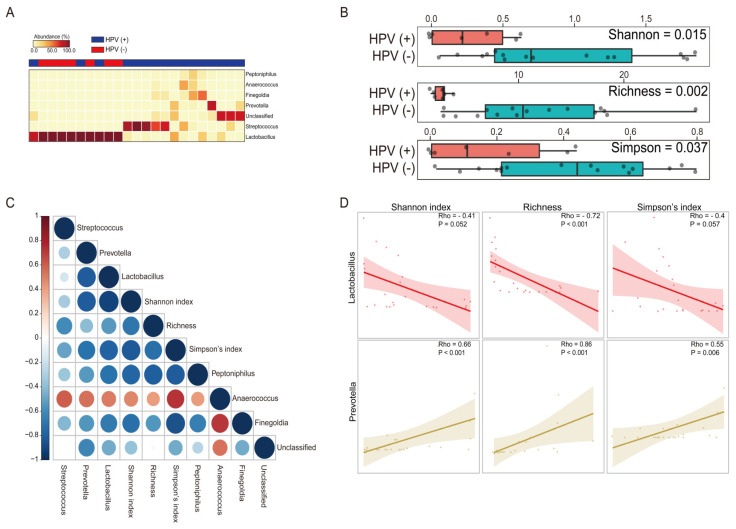
Vaginal microbiota composition and their correlation pattern analysis comparing HPV positive to negative. (**A**) Heat map of the differentially abundant microbiota profile between HPV positive and negative (Wilcoxon sum test; only the genera with *p* < 0.05 were selected). (**B**) Alpha diversity including the Shannon index, Richness, and Simpson’s index is plotted for participants with HPV negative (pink) and positive (green). (**C**) Spearman correlation coefficients (Rho) are presented red for positive correlations and blue for negative correlations. The intensity of the color and size of the dot are associated with the Rho coefficient strength. (**D**) Scatter plots with illustration of the correlation between the specific bacterial genus and alpha diversity of each group’s vaginal microbiota.

To further investigate if HPV infection alters the microbial structure in the vaginal environment, we performed a comparative analysis according to HPV infection status. Comparing the taxonomic composition, we observed significant differences at the genus level. Briefly, our results revealed that women with HPV (+) exhibited higher levels of six genera (*Streptococcus*, *Prevotella*, *Peptoniphilus*, Unclassified, *Finegolida*, and *Anaerococcus*) and a lower level of *Lactobacillus*; in particular, *Lactobacillus* (*p* < 0.001) and *Prevotella* (*p* = 0.002) were two of the most significant genera (Figure 4A). We then computed the alpha diversity and richness to examining their microbial community (Figure 4B). The difference in diversity indexes (Shannon, Richness, and Simpson) with respect to the HPV infection was statistically significant (*p* = 0.015, *p* = 0.002, and *p* = 0.037, respectively) in contrast to a previously reported study [31]. These results imply that possible interplay between HPV infection, the presence of specific bacteria, and diversity of vaginal microenvironment. To investigate the potential interplay between those genera and the diversity of the vaginal microbiota as we described earlier, we conducted a correlation analysis (Figure 4C). Notably, strong correlations were found between the specific microbes and diversity indexes (Figure 4D). For example, *Lactobacillus* negatively correlated to all of diversity indexes with statistical significance (Rho = −0.41, *p* = 0.052; Rho = −0.72, *p* < 0.001; and Rho = −0.4, *p* = 0.057, respectively). Conversely, *Prevotella* showed a significant positive correlation (Rho = 0.66, *p* < 0.001; Rho = 0.86, *p* < 0.001; and Rho = 0.55, *p* = 0.006, respectively). These findings suggest that the status of microbial structure in terms of higher diversity might be regarded as a crucial factor and underlining the importance of specific microbes for HPV infection.

## 4. Discussion

Human microbial profiling offers a variety of understanding of the complicated interplay between the host and microbes in several diseases [32,33,34,35,36,37]. In addition, dysbiosis of microbial composition is potentially considered biomarkers of cancer [38,39]. A previous research performed on human subjects suggested microbial markers of women’s cervical cancer from fecal samples [40]. In contrast to the many studies investigating the impact of HPV infection on the incidence of cervical cancer [41,42,43], whether vaginal microbiota-derived bacteria may be used as a diagnostic tool has not been actively studied yet. Therefore, we aimed to evaluate the potential association between the microbial community and cervical carcinogenesis in Korean cohort. The major result of our preliminary research showed that specific microbes predict the patients in each stage and reflect microbial difference according to the disease severity. On the basis of the clinically confirmed samples and by using a noninvasive biomarker identification analysis (ROC analysis), we discovered three potential candidates as biomarkers, namely, *Lactobacillus*, *Gardnerella*, and *Prevotella* with robust prediction capacity (AUC > 0.8) to distinguish the patients with CIN from healthy participants. Moreover, we identified additional biomarkers to discriminate ICC from CIN (*Gardnerella* or *Streptococcus* (AUC > 0.9)).

It is well demonstrated that a decrease in microbial diversity is observed in healthy vaginal microenvironments, whereas microbial community with greater bacterial diversity with the dominance of facultative and strict anaerobes is a major characteristic of vaginal dysbiosis. From this perspective, the result of this study contains consistent observations regarding the association between bacterial diversity and advanced CIN severity. In a previous study by Mitra et al. [20], vaginal microbiota analysis for 169 healthy women, low-grade squamous intraepithelial lesions, high-grade squamous intraepithelial lesions, and ICC revealed that increased microbiome diversity significantly correlated with CIN severity. Fredricks et al. [44] observed that participants with BV had increased diversity indexes (*p* < 0.001), implying a role of the microbial community in terms of ecological imbalance. In addition, several studies conducted previously also revealed the potential association between the increased vaginal diversity and reproductive tract, such as sexually transmitted infections and preterm birth [45,46]. Similar to the findings of those studies, we showed an increasing trend of the diversity indexes after the disease progression, although statistical significance was not reached between CIN and CAN.

Evidence are increasingly reported that the presence and infection of certain bacteria are one of the most influential factors for the association and progression of various malignancies, including oral cancer [47,48,49]. This study showed that specifically, *Streptococcus* is a potential biomarker that is discriminatory of CAN, likely involving the activation of multiple inflammatory cytokines, and may affect human vaginal and cervical epithelial cells. Patras et al. [50] also supported the immuno-response of the genus by reporting interleukin-17 production in response to the colonization of Group B Streptococcus (GBS). Furthermore, Soares et al. [51] revealed that GBS possesses metallopeptidases that can cleave extracellular matrix proteins composed of fibronectin, laminin, type IV collagen, fibrinogen, and albumin, which may help them invade tissue or cause bacterial dissemination. Conversely, *Lactobacillus* spp. are regarded as the most common vaginal bacteria in the reproductive tract of healthy women. It is well known that this genus produces lactic acid to kill other bacteria and inhibit their binding to epithelial cells [52]. The lactic acid produced by *Lactobacillus* enhances DNA repair and gene expression by blocking histone deacetylases (HDAC) [53]. *Gardnerella* spp. (mainly *G. vaginalis*) is considered one of the main pathogens against vaginal health [54], and our study supports a previous research that prospectively demonstrated that the presence of the genus and progression to precancer are mediated by subsequently increasing vaginal microbial diversity. In a previous study, Usyk et al. [55] reported a positive association between *Gardnerella* and CIN 2+ progression caused by elevated microbial diversity based on multivariate mediation analysis in their longitudinal cohort. Likewise, we found that *Gardnerella* was significantly enriched in the CIN group and further evaluated its potential role as biomarkers. Taken together, it is plausible that proposed biomarkers are potentially associated with disease progression.

In summary, this study aimed to make and evaluate prediction models based on the potential association between vaginal-microbiota-derived microbes and CC in Korean women. The results of this study imply that the presence of specific vaginal microbes are possibly associated with CC. Although testing of HPV infections as a primary screening for ICC is broadly used, most HPV infections are innocuous. Therefore, it is essential to develop additional screening methods to identify women at risk with cervical precancerous lesions and help clinicians in decision making.

We further acknowledge the major limitations associated with our study. First, the cohort size was too small to evaluate the environmental factors which might influence the structure of vaginal microbiota and this might have caused an overestimation or underestimation of the prediction models proposed in this study. Therefore, additional studies are essential to validate the models in a larger cohort. Second, because the results of this study only provide preliminary association between vaginal microbes and health status, caution should be observed when generalizing our findings. Lastly, this research cannot address the causality between vaginal microbiome and disease progression. Thus, clinical research is warranted to reveal how microbes interact with the host. Nonetheless, the observation of this study regarding vaginal microbiota confirms that the dominance of specific microbes may play a potential role in the CC. Furthermore, our study implies that expanding the knowledge on CC-specific bacterial signatures may provide opportunities to investigate the vaginal-microbiota-based prediction and prevention of CC in terms of diagnosis.

## 5. Conclusions

In this study, we aimed to explore differences in microbial diversity and composition according to disease severity, which led to the identification of microbial fingerprints that might be potentially utilized as a noninvasive diagnostic tool in this cohort. Nonetheless, additional studies are warranted to unravel how these bacterial genera interplay with the vaginal environment in terms of causal relationships.

## Figures and Tables

**Table 1:** Characteristics of the study subjects.

Variable	HC (n = 7)	CIN (n = 8)	CAN (n = 8)
Age (years)	47.4 ± 5.38	43.4 ± 12.8	47 ± 10.2
Menopause (n, %)	2 (28.6)	3 (37.5)	2 (25.0)
Marriage (n, %)	5 (71.4)	6 (75.0)	7 (87.5)
Parity (n)	1.1 ± 1.0	1 ± 0.9	1.8 ± 0.8
Smoker (n, %)	0 (0.0)	4 (50.0)	3 (37.5)
Contraceptive use (n, %)	0 (0.0)	3 (37.5)	2 (25.0)
HPV positive (n, %)	0 (0.0)	7 (87.5)	8 (100.0)
HPV16/18 positive (n, %)	0 (0.0)	0 (0.0)	5 (62.5)

## Data Availability

The raw sequences generated for this study are available from the NCBI BioProject under accession numbers PRJNA692362.
